# An Improved Multi-Objective Grey Wolf Optimizer for Bi-Objective Parameter Optimization in Single Point Incremental Forming of Al1060 Sheet

**DOI:** 10.3390/ma19030616

**Published:** 2026-02-05

**Authors:** Xiaojing Zhu, Xinyue Zhang, Jianhai Jiang, Xiaotao Wu, Shenglong Liao, Jianfang Huang, Yuhuai Wang

**Affiliations:** 1 School of Mechanical & Electrical Engineering, China Jiliang University, Hangzhou 310018, China; 2 School of Engineering, Hangzhou Normal University, Hangzhou 310018, China; 3 Zhejiang Machinery Industry Federation, Hangzhou 310009, China

**Keywords:** single point incremental forming, process parameters, multi-layer perceptron, multi-objective grey wolf optimization, entropy-weighted TOPSIS

## Abstract

To address the issues of excessive sheet metal thinning and geometric deviation in single point incremental forming (SPIF), this paper proposed a bi-objective process parameter optimization framework for Al1060 sheet based on a multilayer perceptron (MLP) surrogate model and an improved multi-objective grey wolf optimization (IMOGWO) algorithm. Finite element simulations based on ABAQUS were conducted to generate a dataset considering variations in tool radius, initial sheet thickness, tool path strategy, step depth and forming angle. The trained MLP was used as the objective function in the optimization process to enable the rapid prediction of forming quality. The IMOGWO algorithm, enhanced by the Spm chaotic mapping initialization, an improved convergence coefficient updating mechanism and associative learning mechanism, was then employed to efficiently search for Pareto optimal solutions. For a truncated conical component case, optimal parameter sets were selected from the Pareto front via the entropy-weighted TOPSIS method for order preference by similarity to an ideal solution. Experimental verification showed close agreement with the simulated results, with relative errors of only 0.58% for the thinning rate and 3.10% for the geometric deviation. This validation demonstrates the feasibility and potential of the proposed method and its practical potential for improving the quality of SPIF forming.

## 1. Introduction

Single point incremental forming (SPIF) [1] is an eco-friendly, flexible sheet metal forming technique derived from the layer-by-layer deposition principle of additive manufacturing. This process employs a numerical control system to precisely guide the forming tool along a predefined trajectory, inducing localized plastic deformation of the sheet material point by point and layer by layer. This cumulative deformation ultimately achieves the efficient fabrication of complex curved components. As it requires no dedicated molds or only simple backing molds during forming, this technology significantly reduces tooling costs and shortens product development cycles. Consequently, it demonstrates unique advantages for manufacturing complex curved components and small-batch customized products, such as personalized medical implants. However, its engineering applications remain severely constrained by limitations including excessive geometric deviation, suboptimal surface quality, and pronounced local thinning of the sheet material [2].

To enhance the effectiveness of SPIF applications, researchers have proposed several improvement measures, including multi-stage forming [3], thermo-assisted processing [4], process variations [5], and process parameter optimization [6]. Among these, multi-stage forming reduces spring-back by applying forming processes with a hardening effect in stages, though it significantly prolongs manufacturing time. Thermally assisted SPIF also reduces spring-back, but requires higher equipment specifications, and is typically only applicable to materials with poor formability at ambient temperatures. Process modifications improve forming quality by reinforcing forming regions (e.g., using counter-supports or partial dies), though this increases equipment costs. In contrast, process parameter optimization enhances forming accuracy and surface quality directly and efficiently by minimizing local thinning and spring-back through optimizing critical forming parameters such as feed rate, tool trajectory, forming angle, and initial sheet thickness. This approach is a highly effective solution.

In practical SPIF applications, various performance indicators, such as thickness reduction, geometric deviation, texture and microstructure [7], are utilized to assess the outcome. However, geometric deviation and thickness reduction are two key indicators that directly determine part usability and structural integrity. Geometric deviation reflects the difference between the formed part and the target geometry, which is a critical quality requirement in industrial forming processes. Thickness reduction, on the other hand, is closely associated with localized necking and fracture risk, representing the primary constraint for preventing failure. The macroscopic performance of both indicators is fundamentally governed by microstructural evolution, particularly the development of deformation texture. More importantly, these two objectives are often mutually exclusive: aggressive parameters aimed at minimizing deviation frequently trigger excessive thinning, whereas conservative settings to preserve thickness may lead to significant spring-back and poor accuracy. Therefore, these two objectives, respectively, represent product quality and forming safety, defining the critical trade-off necessary for optimizing the SPIF process. Extensive work has been conducted on optimizing process parameters for these objectives. For instance, Habeeb et al. [8] experimentally and statistically demonstrated that increasing step depth appropriately reduces thickness reduction while enhancing forming quality. Samad et al. [9] achieved an accuracy of 92% in predicting the thickness distribution using a random forest model. Yang et al. [10] utilized deep learning models to predict spring-back and reduce geometric deviation. Najm et al. [11] achieved the rapid prediction and optimization of geometric deviation through artificial neural networks (ANNs). However, multiple process parameters in SPIF interact synergistically to influence forming quality. Single-objective optimization typically compromises other performance metrics. For instance, optimizing the thinning rate to enhance material utilization may compromise the forming geometric accuracy. Coordinating multiple process parameters to optimize metrics such as thinning rate and geometric deviation simultaneously constitutes a complex multi-objective optimization problem. Traditional optimization methods, such as genetic algorithm (GA), are inefficient when addressing such multi-objective problems, as they struggle to locate global optima within reasonable timeframes. To achieve rapid convergence towards global optima, Moses et al. [12] proposed an enhanced squirrel search algorithm. This approach optimized seven SPIF process parameters, effectively reducing geometric deviations and enhancing forming accuracy. Experimental results demonstrate that truncated cones formed using the optimized SPIF process exhibit root mean square errors of 2.4 mm^2^ for roundness deviation and 3.2 mm^2^ for positional deviation. This highlights the importance of developing more efficient optimization algorithms for enhancing both SPIF multi-objective optimization and overall forming performance.

Multi-objective grey wolf optimization (MOGWO) [13], proposed by Mirjalili et al. in 2016, is a novel meta-heuristic optimization method that simulates the hunting behavior of grey wolf packs in nature. This algorithm mathematically models the social hierarchy and behaviors of wolves, such as encircling, pursuing, and attacking prey. It effectively combines global search with local exploration. It features minimal parameters and a straightforward structure. Its leader-follower guided search strategy effectively prevents premature convergence, enhances convergence speed, and strengthens the algorithm’s directionality towards finding optimal solutions, thereby accelerating the discovery of the global optimum [14]. Existing research confirms that MOGWO significantly outperforms traditional algorithms such as NSGA-II and multi-objective particle swarm optimization in terms of convergence and solution set distribution quality, demonstrating particular advantages in complex optimization problems [15]. Although MOGWO has been extensively applied in various fields, including energy system optimization [16], logistics route optimization [17], and parameter tuning [18], its utilization in SPIF parameter optimization is scarcely documented. Consequently, applying MOGWO to SPIF parameter optimization is a promising avenue for further research.

The application of MOGWO for SPIF parameter optimization requires a suitable objective function [19]. Due to its robust nonlinear modeling and multivariable coupling capabilities, the neural network has become a common choice for constructing SPIF parameter optimization objective functions [20]. Unlike traditional statistical methods, neural networks do not rely on low-order polynomial assumptions, significantly enhancing prediction accuracy and model generalization performance. For instance, Ajay et al. [21] achieved a correlation coefficient of 0.99992 by using an ANN to optimize wall angles and surface roughness for Ti-Grade 5 material. Kumar et al. [22] proposed a hybrid artificial neural network to estimate the maximum forming load for AA7075-O sheet, attaining a prediction accuracy of 99.80%. Regarding multi-objective co-optimization, Xiao et al. [23] proposed an AA5052 SPIF forming optimization method based on a back propagation neural network and GA, achieving both maximized forming angle and minimized thinning rate while obtaining a Pareto optimal solution. Taherkhani et al. [24] proposed a synergistic optimization method for SPIF geometric accuracy and surface quality based on the group method of data handling and GA. It simultaneously enhances geometric accuracy and surface quality, achieving a minimum geometric deviation of 0.79 mm and a surface roughness of 0.57 mm. Consequently, developing a neural network agent model for SPIF multi-objective optimization can enhance optimization accuracy.

Multi-objective parameter optimization problems are characterized by strong parameter coupling, narrow feasible regions, and conflicting quality objectives, which make conventional multi-objective metaheuristic algorithms prone to premature convergence and poorly distributed Pareto fronts. Existing studies often focus on improving a single aspect of the optimization process, such as population diversity, convergence speed, or leader update strategies [25,26,27,28]. In particular, dynamic ensemble-learning models have incorporated GWO variants for complex risk assessment and high-level predictive modeling [25], primarily for structural reliability analysis and dynamical weighting optimization. Nevertheless, in complex practical problems such as SPIF parameter optimization, the search space is continuous, highly coupled, and severely constrained. Under these conditions, existing variants of metaheuristic algorithms often struggle to maintain solution diversity within narrow feasible regions and fail to simultaneously ensure effective global exploration capability, convergence stability, and a well-distributed Pareto front.

To achieve synergistic optimization of the SPIF thinning rate and geometric deviation, this paper proposed a dual-objective optimization method combining MLP and improved multi-objective grey wolf optimization (IMOGWO). First, a sample dataset was constructed using finite element simulations, and an MLP neural network was established and trained to create a nonlinear mapping model of thinning rate and geometric deviation with respect to forming process parameters. Subsequently, an IMOGWO variant was proposed to enhance the diversity of algorithmic initialization and the exchange of information within grey wolf populations, thereby improving convergence efficiency and global optimization capability. This was achieved through chaotic mapping initialization, an improved convergence coefficient updating mechanism and a cooperative learning mechanism. Finally, the Pareto optimal solution set was screened for optimal process parameters using the entropy-weighted TOPSIS method. The finite element simulation and experimental results of the truncated cones made from Al 1060 sheet metal are consistent, thus verifying the effectiveness of the proposed IMOGWO method.

## 2. Finite Element Simulation and Dataset Establishment

To obtain a high-quality sample set for data-driven, precise prediction of forming performance and parameter optimization, SPIF finite element simulations of the truncated conical components were implemented based on ABAQUS/Explict. The required dataset for optimization was then constructed using the extracted thinning rate and the geometric deviation, presenting the forming accuracy.

### 2.1. Selection of SPIF Process Parameters and Performance Evaluation Metrics

**Figure 1:**
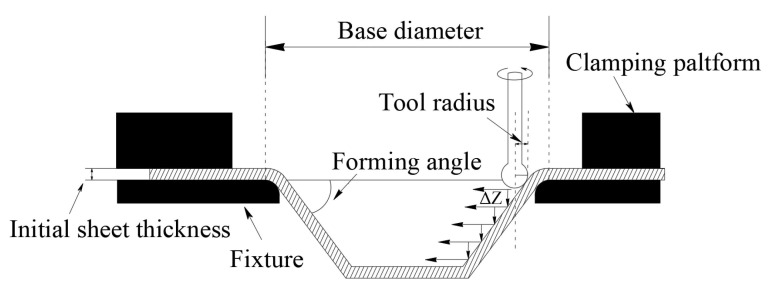
SPIF process for a truncated cone.

The SPIF process for a truncated cone is illustrated in Figure 1. The key parameters that influence the performance of the SPIF forming process include the tool radius, the initial sheet thickness, the tool path strategy, the step depth and the forming angle. Common levels for these process parameters are shown in Table 1.

The final sheet thickness after SPIF forming can be estimated using the sine rule [29], as shown in Equation (1):

(1) $$t=t_{0}\cdot \sin\left(\frac{\pi }{2}-\theta \right)=t_{0}\cdot \cos\theta$$

where $t_{0}$ and t denote the initial and final sheet thickness, respectively. θ is the forming angle.

To produce high-quality formed components with uniform thickness distribution and high-profile accuracy, the thinning rate and geometric deviation are selected as the two key metrics used to evaluate forming performance. The thinning rate quantifies the extent of thickness variation during the SPIF process and serves as a critical indicator of forming uniformity and structural integrity. In industrial applications, the thinning rate is closely associated with defect occurrence and the structural reliability of the final product, as values exceeding a critical threshold can induce localized necking or even tearing. Geometric deviation, on the other hand, characterizes the discrepancy between the formed profile and the nominal design geometry and constitutes a primary criterion for evaluating forming accuracy and overall quality. From an engineering standpoint, minimizing geometric deviation is essential to ensure assembly compatibility, particularly in high-precision manufacturing scenarios. Moreover, improved geometric deviation reduces the need for energy-intensive secondary post-processing and manual calibration, thereby lowering production costs and reducing energy consumption. The maximum thinning rate, $\eta_{\max}$, and the maximum geometric deviation, $L_{\max}$, which characterize the most critical node in terms of thickness reduction and geometric deviation, respectively. They can be expressed by Equation (2):

(2) $$\left\{\begin{array}{l} \eta_{\max}=\underset{i\in [1,N]}{\max}\frac{\left|t_{i}-t_{0}\right|}{t_{0}}\times 100\%\\ L_{\max}=\underset{i\in [1,N]}{\max}\left|l_{i}\right| \end{array}\right.$$

where N represents the total number of measurement points on the sheet in the test results. $t_{i}$ and $l_{i}$ denotes the thickness and deviation between the actual and ideal profiles, at the *i*-th measurement point, respectively.

Other SPIF performance indicators, such as surface roughness and forming limit proximity, are also important. However, surface roughness is strongly influenced by tool path strategy, lubrication conditions, and step size, and is typically optimized in a separate process planning stage. Restricting the optimization problem to two objectives does not oversimplify the SPIF process. Instead, it reflects the minimal set of objectives necessary to balance the part quality and structural stability. Introducing additional objectives would increase the complexity of optimization without necessarily improving practical applicability.

### 2.2. Test Material Properties

This study employed Al1060 alloy sheet, the chemical composition of which is detailed in Table 2. With an aluminum content of up to 99.6%, this material exhibits excellent plastic deformation capabilities and favorable isotropic properties, making it suitable for the SPIF process.

**Figure 2:**
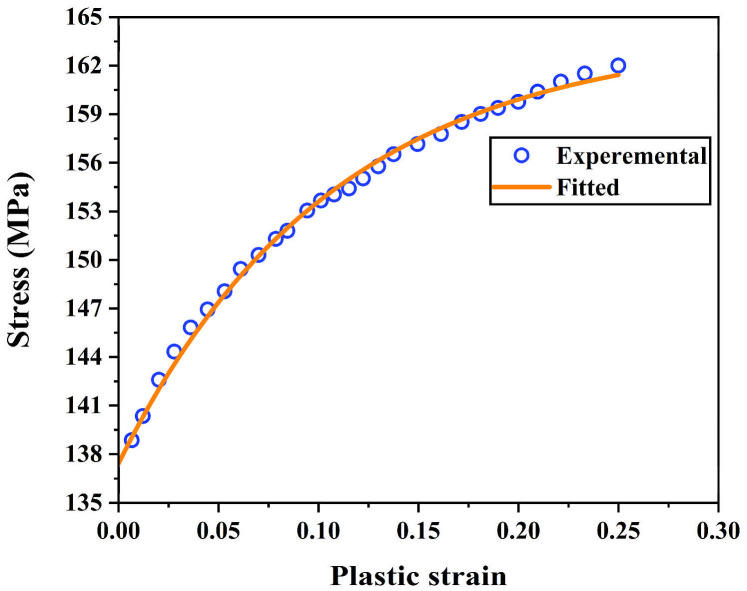
The extracted true stress-true plastic strain data and the Voce model fitting curve for Al1060.

To determine the mechanical properties of Al1060, uniaxial tensile tests were conducted on dumbbell-shaped plate specimens using a computer-controlled electronic universal testing machine (WDW-50C, Shanghai Hualong Testing Instrument Co., Ltd., Shanghai, China; accuracy class 0.5) in accordance with the ASTM E8/E8M standard [30]. The testing machine provided the load–displacement data. The engineering stress–strain curves were calculated from these measurements, and the experimental data points shown in Figure 2 were subsequently extracted and converted into true stress-true plastic strain data for constitutive model fitting. Considering that Al1060 will undergo significant plastic strain during the SPIF process, the Voce hardening model [31], which can accurately describe the hardening behavior of the material across a large strain range, was used to fit the nonlinear relationship between true stress, σ, and true plastic strain, $\varepsilon_{p}$, for Al1060, as shown in Equation (3):

(3) $$\sigma =\sigma_{0}\left(1-Ae^{-B\varepsilon_{p}}\right)$$

where $\sigma_{0}$ denotes the saturation flow stress, B representing the material’s hardening index, A denotes the hardening rate coefficient. The true stress–true plastic strain data and corresponding Voce fit for Al1060 are shown in Figure 2, focusing on the plastic region where the model describes flow stress hardening. Young’s modulus was obtained from the initial linear segment of the engineering stress–strain curve. With a coefficient of determination of *R*^2^ = 0.9960, the established Voce constitutive model can be considered to accurately describe the work hardening behavior of Al1060 during plastic deformation.

Table 3 presents the primary physical and mechanical properties of Al1060, which will be used for subsequent finite element simulations.

### 2.3. Finite Element Modeling and Simulation

**Figure 3:**
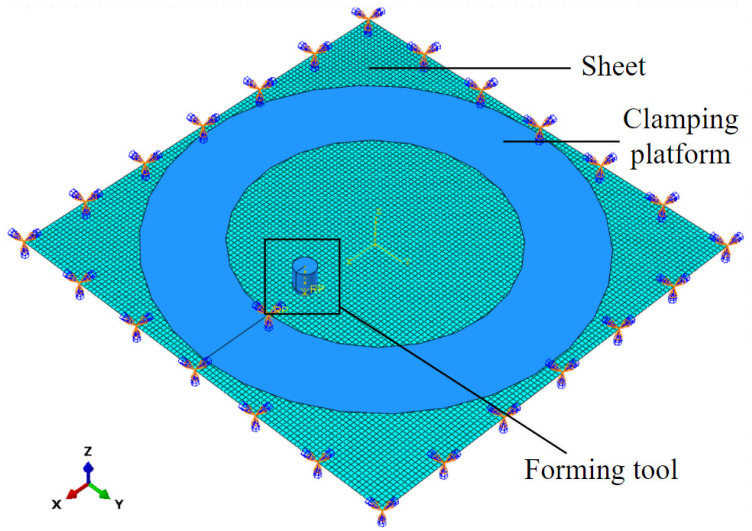
The developed SPIF finite element model of the aluminum sheet.

Finite element simulations of the truncated conical components were carried out using the Abaqus/Explicit platform with Al1060 sheets. In the simulation, the forming tool and the clamping platform were modeled as rigid bodies, whereas the sheet was modeled as a deformable body. The aluminum sheet with a side length of 200 mm was modeled as a square sheet with fully fixed constraints on all four edges. The coefficient of friction at the contact surfaces was set to 0.1. A mesh comprising 4-node reduced integral elements (S4R) for explicit dynamics analysis and 3-node triangular elements (S3R) for mesh transition regions was employed. The global seed size was set to 2.0 mm. The developed SPIF finite element model of the aluminum sheet, shown in Figure 3, consists of 10,000 quadrilateral shell elements and 10,201 nodes.

To enhance the predictive accuracy of subsequent neural network models, a full factorial experimental design was implemented according to the process parameters outlined in Table 1. Parametric driving was achieved through Python 3.10 scripting, enabling finite element simulation of the SPIF for circular tapered aluminum sheet sections under 718 distinct process parameter combinations. By extracting the thickness distribution and displacement field data of the truncated conical components post-forming, the corresponding maximum thinning rate and maximum geometric deviation for each dataset were calculated using Equation (2). This established a sample dataset for predicting thinning rate and geometric deviation.

To ensure the reliability of the dataset, data curation was conducted to verify simulation integrity. Each of the 718 process parameter combinations was checked for numerical stability and physical consistency before being utilized for MLP training.

## 3. SPIF Performance Prediction Model Based on MLP

To rapidly and accurately predict the forming performance of SPIF, this study established an MLP neural network model for predicting the forming thinning rate and geometric deviation based on the SPIF dataset constructed in Section 2. This provides a foundation for implementing subsequent optimization algorithms.

### 3.1. MLP Neural Networks

**Figure 4:**
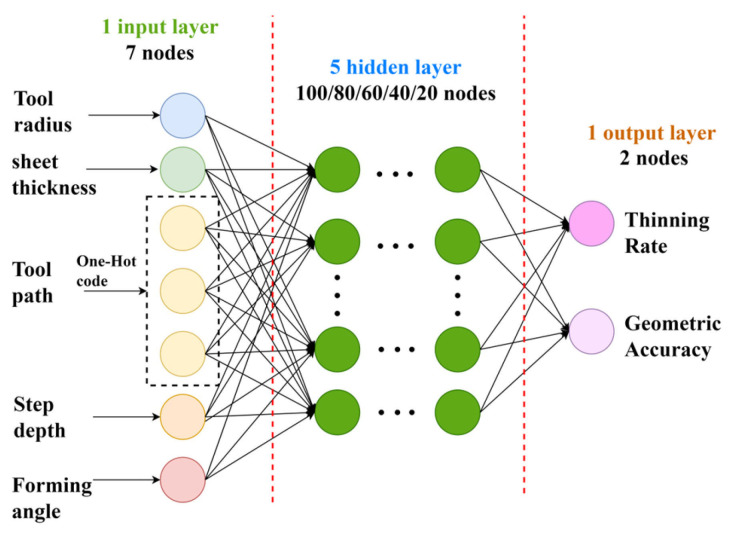
The constructed MLP neural network architecture diagram.

The designed six-layer MLP neural network architecture is illustrated in Figure 4. The input layer comprises five process parameters: tool radius, initial sheet thickness, tool path strategy, forming angle, and step depth. Considering the three forming strategies—contour path, helical path and reverse contour path as categorical inputs, one-hot encoding was employed to convert them into distinct binary categories. Consequently, the input layer comprises seven neurons. The two output layer neurons represent the thinning rate and geometric deviation, respectively. There are 100, 80, 60, 40 and 20 neurons in the five hidden layers, respectively. The tanh(*x*) function, as shown in Equation (4), was used as the activation function.

In SPIF modeling, the mapping from process parameters to thickness reduction and geometric deviation exhibits strong nonlinearity and parameter coupling. Preliminary tests showed that shallow networks tend to underfit this relationship, resulting in reduced prediction accuracy, particularly near the feasible boundary of the process window. The proposed MLP architecture contains multiple hidden layers relative to the dataset size. The network depth is not selected arbitrarily, but is intended to capture the highly nonlinear and strongly coupled relationship between SPIF process parameters and forming responses.

(4) $$\tanh(x)=\frac{1-\exp\left(-2x\right)}{1+\exp\left(-2x\right)}$$

To prevent overfitting and enhance the model’s generalization capability, the dropout technique [32] and L2 regularization were incorporated during network training. The dropout rate was set to 0.2, meaning that, during training, 20% of the hidden layer neurons and their connections were randomly discarded, thereby mitigating co-adaptation phenomena between neurons. The weight decay coefficient for L2 regularization was set to 0.001 to penalize excessively large network weights and suppress model complexity. The synergistic effect of these two regularization methods effectively mitigated the risk of overfitting within the relatively limited dataset for the SPIF modeling scenario. This ensured that the constructed MLP model was highly accurate and stable, and less prone to overfitting.

### 3.2. MLP Training and Performance Evaluation

**Figure 5:**
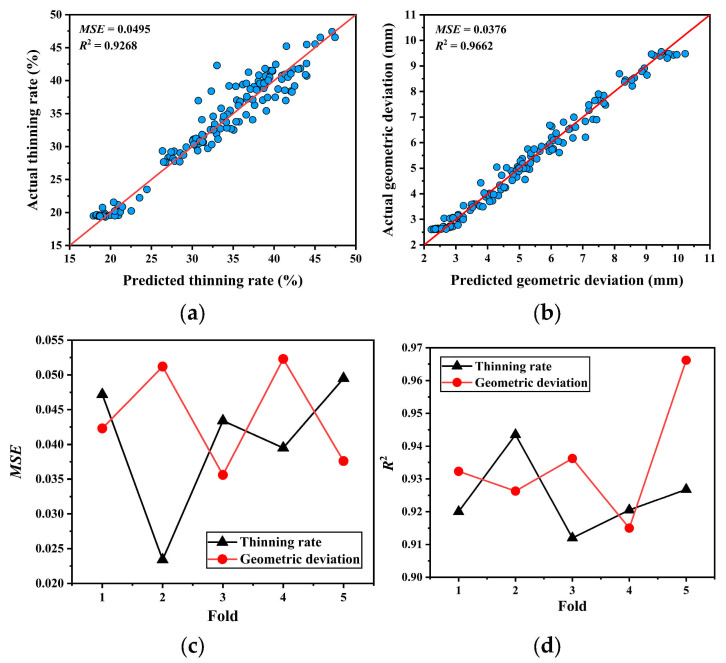
The performance results of the MLP model obtained through five-fold cross-validation: (**a**) thinning rate prediction results of the fifth fold; (**b**) geometric deviation prediction results of the fifth fold; (**c**) *MSE* variation during five-fold training; (**d**) *R*^2^ variation during five-fold training. The blue dots represent individual test samples, while the red line denotes the ideal y = x reference line indicating perfect prediction.

During training, the batch size, number of epochs, and learning rate were set to 32, 1000, and 0.0001, respectively. The Adam optimizer and mean squared error loss function were employed. To evaluate the model’s robustness and generalization capability, a five-fold cross-validation strategy was adopted. The mean squared error, *MSE*, and the coefficient of determination, *R*^2^, were employed, as shown in Equation (5), to assess the model’s predictive accuracy and reliability.

(5) $$\left\{\begin{array}{l} MSE=\frac{1}{n}\sum_{i=1}^{Z}{\left(Y_{i}-{\hat{Y}}_{i}\right)}^{2}\\ R^{2}=1-\frac{\sum_{i=1}^{Z}{\left(Y_{i}-{\hat{Y}}_{i}\right)}^{2}}{\sum_{i=1}^{Z}{\left(Y_{i}-\frac{1}{Z}\sum_{i=1}^{Z}{\hat{Y}}_{i}\right)}^{2}} \end{array}\right.$$

where ${\hat{Y}}_{i}$ denotes the predicted value for the *i*-th sample in the test set, and $Y_{i}$ denotes the corresponding true value. Z indicates the total number of samples in the test set. Figure 5 shows the performance results of the MLP model obtained through five-fold cross-validation. Figure 5a and Figure 5b show the comparison between the predicted and actual values for thinning rate and geometric deviation, respectively, on the validation set using the MLP trained on the fifth fold. As can be seen from the figures, the predictions from the MLP trained on the fifth fold closely align with the actual values on the validation set, demonstrating the accuracy of the training process.

Figure 5c and Figure 5d show the variations in the *MSE* and *R*^2^, respectively, on the validation set for the MLP trained on each fold of the dataset. As can be seen in Figure 5c,d, the changes in both *MSE* and *R*^2^ metrics remained stable throughout the training and validation processes. The *MSE* remained consistently below 0.055, indicating high training accuracy and generalization capability of the MLP model, with no discernible signs of overfitting. Concurrently, *R*^2^ exceeded 0.93, demonstrating strong alignment between the predicted and actual values. This further confirms the high precision and reliability of the trained MLP.

The MLP model serves as a surrogate representation of the SPIF process rather than an exact physical model. The prediction accuracy of the surrogate model is therefore critical to ensuring the reliability of the subsequent optimization results. In five-fold cross-validation, the consistently low *MSE* values and high *R*^2^ scores across all folds indicate stable generalization performance and low sensitivity to training data partitioning, suggesting that the surrogate model can reliably capture the nonlinear relationship between process parameters and forming outcomes.

To justify the selected MLP architecture, three alternative models, a deep-narrow MLP (20, 20, 20, 20, 20), a shallow-wide MLP (100, 60, 20) and a shallow-narrow MLP (20, 20, 20), were evaluated using five-fold cross-validation.

**Figure 6:**
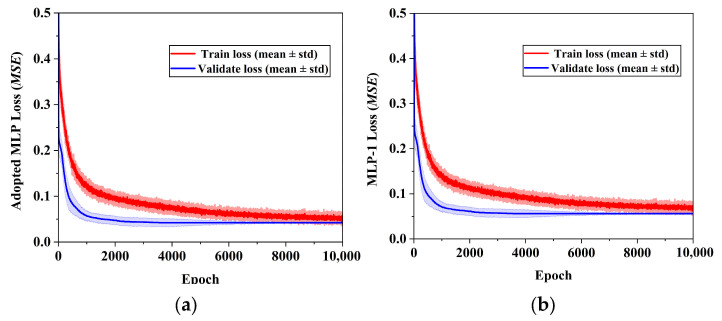

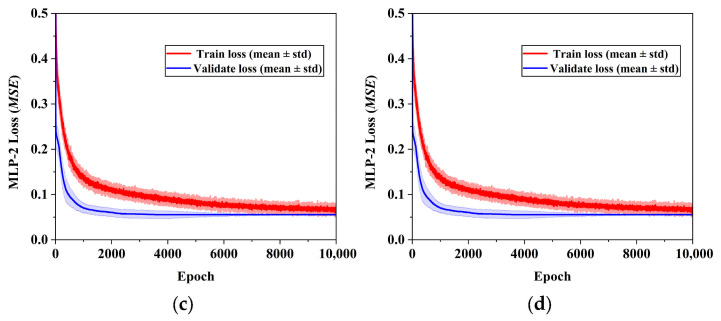
Mean training and validation loss curves of different MLP architectures obtained via five-fold cross-validation: (**a**) Adopted MLP (100, 80, 60, 40, 20); (**b**) MLP-1 (20, 20, 20, 20, 20); (**c**) MLP-2 (100, 60, 20); (**d**) MLP-3 (20, 20, 20). The shaded regions indicate ±1 standard deviation across folds.

Figure 6 presents the mean training and validation loss curves of different MLP architectures obtained via five-fold cross-validation, with shaded bands representing ±1 standard deviation across folds. All models exhibit rapid loss reduction during the early training stage followed by gradual convergence, indicating stable optimization behavior. The relatively narrow standard deviation bands demonstrate good consistency among different folds. Moreover, the small gap between the training and validation loss curves indicates that overfitting is effectively controlled and the models possess satisfactory generalization performance.

**Figure 7:**
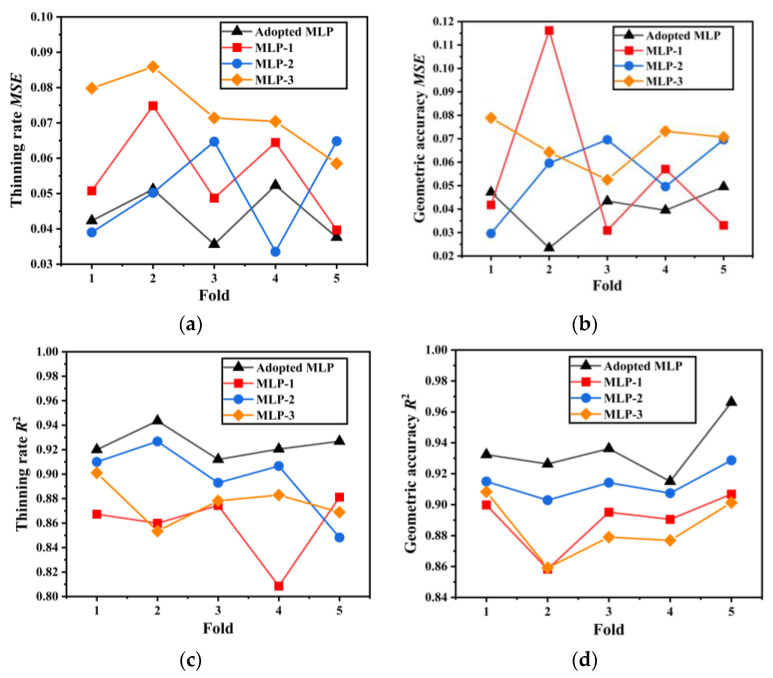
The performance comparison of the four MLP models under five-fold cross-validation: (**a**) thinning rate *MSE*; (**b**) geometric deviation *MSE*; (**c**) thinning rate *R*^2^; (**d**) geometric deviation *R*^2^.

Table 4 summarizes the mean validation *MSE* and *R*^2^ for both thinning rate and geometric deviation. In addition, Figure 7 illustrates the fold-wise variation in *MSE* and *R*^2^ for the four MLP models. The adopted MLP achieves lower validation *MSE* and higher *R*^2^ values across most folds, indicating superior generalization performance. The deep-narrow architecture suffers from limited representational capacity, while the shallow-wide architecture shows less stable cross-validated performance. In contrast, the adopted MLP provides a balanced depth-width configuration, leading to more reliable generalization under limited data conditions.

## 4. SPIF Multi-Objective Optimization Based on IMOGWO

The GWO models the parameter optimization process as the hunting behavior of grey wolves [33]. In this algorithm, each grey wolf represents a potential solution to the parameter optimization problem. The three wolves with the highest fitness are designated as the Alpha, Beta, and Delta wolves, forming the pack of head wolves. The head wolves then guide the pack’s search behavior, steering it towards the optimal solution.

To apply GWO to multi-objective optimization problems, Mirjalili et al. [13] introduced the Pareto front to record optimal solutions and employed a roulette wheel selection method for selecting head wolves, thereby constructing the MOGWO algorithm. However, traditional MOGWO still suffers from issues such as getting stuck in local optima and slow convergence. Therefore, an IMOGWO algorithm was presented and investigated to achieve the co-optimization of the SPIF thinning rate and geometric deviation based on the MLP model established in Section 3 as the cost function. By incorporating the Spm chaotic initialization, an improved convergence coefficient, and an enhanced wolf pack associative learning mechanism, MOGWO was refined to address its susceptibility to local optima and slow convergence. This improves the accuracy and speed of the SPIF parameter optimization process [34]. Unlike existing MOGWO variants that enhance a single optimization aspect, the proposed IMOGWO adopts a coordinated and stage-aware design to address the coupled, constrained, and conflicting nature of SPIF multi-objective optimization.

### 4.1. Spm Chaos Mapping Initialization

Traditional MOGWO typically employs random population initialization methods, which can readily lead to uneven initial population distributions, causing the algorithm to converge to local optima. To address this, this paper introduces Spm chaotic mapping initialization [35], which enables the initial distribution of the wolf pack to exhibit superior exploration and randomness. This enhances the algorithm’s ability to explore unknown spaces and improves its global performance. Concurrently, Spm chaotic mapping initialization positions the initial population closer to optimal solutions, accelerating the convergence of the IMOGWO algorithm.

In the SPIF parameter optimization probe, continuous parameter variables can be initialized as the Spm chaotic mapping:

(6) $$X_{i}=(X_{ub}-X_{lb})\chi_{i}+X_{lb}$$

where $X_{i}$ is the initialized parameters for the *i*-th grey wolf within the IMOGWO, $X_{lb}$ and $X_{ub}$ represent the lower and upper bounds, respectively, for the values of the parameters to be optimized. The Spm chaos mapping initialization factor, $\chi_{i}$, can be given as follows:

(7) $$\chi_{i}=\left\{\begin{array}{l} \begin{array}{cc} \mathrm{mod}\left(\frac{\chi_{i-1}}{\gamma }+\psi \sin\left(\pi \chi_{i-1}\right)+\lambda_{i},1\right), & 0<\chi_{i-1}\leq \gamma \end{array}\\ \begin{array}{cc} \mathrm{mod}\left(\frac{\chi_{i-1}/\gamma }{0.5-\gamma }+\psi \sin\left(\pi \chi_{i-1}\right)+\lambda_{i},1\right), & \gamma <\chi_{i-1}\leq 0.5 \end{array}\\ \begin{array}{cc} \mathrm{mod}\left(\frac{1-\chi_{i-1}/\gamma }{0.5-\gamma }+\psi \sin\left(1-\chi_{i-1}\right)+\lambda_{i},1\right), & 0.5<\chi_{i-1}\leq 1-\gamma \end{array}\\ \begin{array}{cc} \mathrm{mod}\left(\frac{1-\chi_{i-1}}{\gamma }+\psi \sin\left(1-\chi_{i-1}\right)+\lambda_{i},1\right), & 1-\gamma <\chi_{i-1}\leq 1 \end{array} \end{array}\right.$$

where $\lambda_{i}$ is a random number uniformly distributed within the interval (0, 1), which serves as the initial seed for the chaotic iterations. mod(.) is the modulo operator, ensuring the generated values remain within the predetermined range. γ and ψ are coefficients controlling the dynamic properties of the mapping, with values between 0 and 1. Here, γ = 0.4 and ψ = 0.3 are used to achieve a relatively uniform and random initial distribution [36].

**Figure 8:**
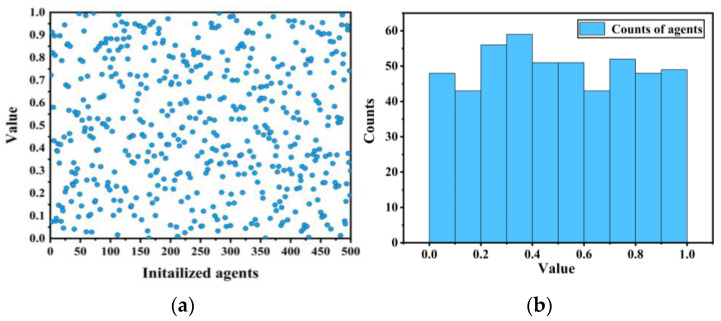
Spm chaos mapping: (**a**) population distribution; (**b**) population size statistics chart. The blue dots represent initialized agents.

When the number of grey wolf agents in IMOGWO reaches 500, the Spm chaotic mapping initialization factors are shown in Figure 8. As can be seen from the figure, the population initialization of the Spm chaotic mapping exhibits good uniformity, effectively enhancing the ability to explore unknown spaces.

### 4.2. Improved Convergence Coefficient

In the MOGWO algorithm, the process of finding the optimal solution to a problem is analogous to a pack of grey wolves searching for or pursuing prey. During the iterations of the algorithm, the behavior of the wolves is influenced by the head wolves, as shown in Equation (8):

(8) $$X_{i}\left(k+1\right)=\frac{1}{3}\left(X_{i}^{\alpha }\left(k+1\right)+X_{i}^{\beta }\left(k+1\right)+X_{i}^{\delta }\left(k+1\right)\right)$$

where $X_{i}\left(k+1\right)$ denotes the potential parameter solution for the *i*-th grey wolf after the (*k* + 1)-th iteration. $X_{i}^{\alpha }\left(k+1\right)$, $X_{i}^{\beta }\left(k+1\right)$ and $X_{i}^{\delta }\left(k+1\right)$ represent the updated parameter solutions for the *i*-th grey wolf, influenced by α, β and δ, respectively, which can be computed using Equation (9).

(9) $$\left\{\begin{array}{l} \begin{array}{cc} X_{i}^{p}\left(k+1\right)=\left(X_{i}\left(k\right)-V\left(k\right)\cdot D_{i}^{p}\left(k\right)\right), & p\in \left\{\alpha ,\beta ,\delta \right\} \end{array}\\ V\left(k\right)=2v\left(k\right)\cdot p_{1}-v\left(k\right)\\ \begin{array}{cc} D_{i}^{p}\left(k\right)=\left|2p_{2}\cdot X_{p}\left(k\right)-X_{i}\left(k\right)\right|, & p\in \left\{\alpha ,\beta ,\delta \right\} \end{array} \end{array}\right.$$

where $X_{p}\left(k\right)$ represents the potential parameter solution indicated by the head wolves after the *k*-th iteration in the IMOGWO. $p_{1}$ and $p_{2}$ are random numbers following a uniform distribution within the interval (0, 1). $v\left(k\right)$ is the convergence coefficient for the *k*-th iteration in the algorithm. It decreases linearly with the iteration number during the search for the optimal solution, controlling the wolves’ actions by influencing $V\left(k\right)$. When $V\left(k\right)>1$, the wolves perform a global search, whereas when $V\left(k\right)\leq 1$, they deploy a coordinated pursuit and converge toward the optimal solution.

**Figure 9:**
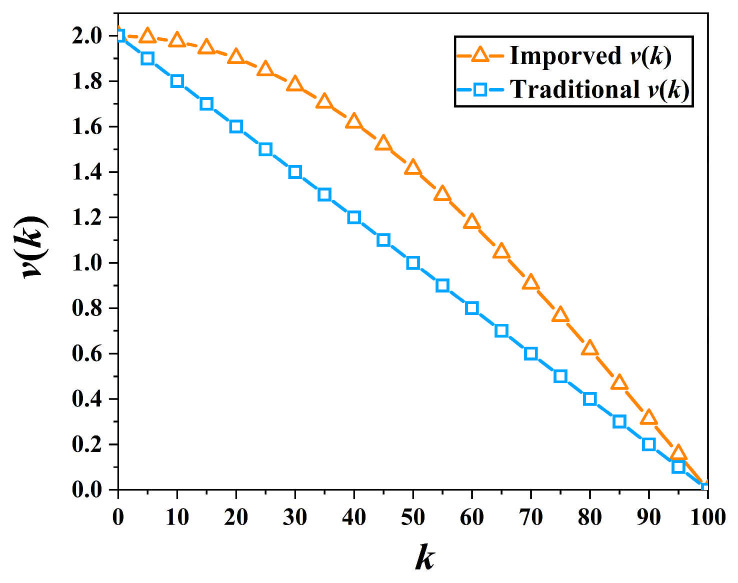
Comparison of improved and traditional $v\left(k\right)$.

To enhance the global search capability of the IMOGWO, the traditional linear descent was replaced with a cosine iteration scheme that constructs a convergence coefficient $v\left(k\right)$ [37], as shown in Equation (10).

(10) $$v\left(k\right)=2\cos\left(\frac{k}{M}\cdot \frac{\pi }{2}\right)$$

where M denotes the maximum number of iterations. The comparison of the $v\left(k\right)$ before and after improvement over the number of iterations is shown in Figure 9. It can be observed that the improved $v\left(k\right)$ decreases slowly during the initial iteration phase and then declines rapidly as the number of iterations increases. Therefore, based on the improved $v\left(k\right)$, IMOGWO maintains global search behavior in the early stages, thereby enhancing the algorithm’s capability for global optimization.

### 4.3. Associative Learning-Based Update Mechanism

In the process of seeking optimal solutions using the traditional MOGWO algorithm, each iterative update is solely influenced by the head wolves. Consequently, the algorithm may fail if the head wolves are trapped in a local optimum. To enhance the global search capability of the algorithm, an associative learning mechanism [26] was introduced to the IMOGWO. The grey wolves on the Pareto front, excluding the alpha wolf, are updated using Equation (11).

(11) $$\begin{array}{cc} X_{i}\left(k+1\right) & =X_{i}\left(k\right)+0.001G\left(X_{i}\left(k\right)-X_{lb},X_{ub}-X_{i}\left(k\right)\right)\\ & \;+\varsigma_{1}w_{1}\left(X_{r}\left(k\right)-X_{i}\left(k\right)\right)+\varsigma_{2}w_{2}\left(X_{\alpha }\left(k\right)-X_{i}\left(k\right)\right) \end{array}$$

where $G\left(X_{i}\left(k\right)-X_{lb},X_{ub}-X_{i}\left(k\right)\right)$ denotes a bounded random perturbation, whose magnitude is adaptively limited by the distances from the current solution to the lower and upper bounds. $w_{1}$ and $w_{2}$ denote random numbers uniformly distributed within the interval (0, 1); $X_{r}\left(k\right)$ denotes the parameters of a random grey wolf from the current Pareto front, excluding the alpha wolf. Equation (9) shows that the update process based on the associative learning mechanism is influenced by other grey wolves in the Pareto front, as well as the alpha wolf. This indicates associative learning behavior among grey wolves. The convergence factors $\varsigma_{1}$ and $\varsigma_{2}$ can be calculated using Equation (12):

(12) $$\left\{\begin{array}{l} \varsigma_{1}=1-\frac{k}{M}\\ \varsigma_{2}=\frac{2k}{M} \end{array}\right.$$

As can be seen from Equation (12), as the iteration number, *k*, increases, $\varsigma_{1}$ decreases while $\varsigma_{2}$ increases. Consequently, the update process of the grey wolf becomes less influenced by random solutions within the Pareto front and more influenced by the alpha wolf. During the initial phase of the algorithm, therefore, the wolves within the Pareto front tend to interact with each other, preventing the algorithm from becoming trapped in local optima. In the later phase, the process converges toward the alpha wolf, ensuring the convergence accuracy of the algorithm.

**Figure 10:**
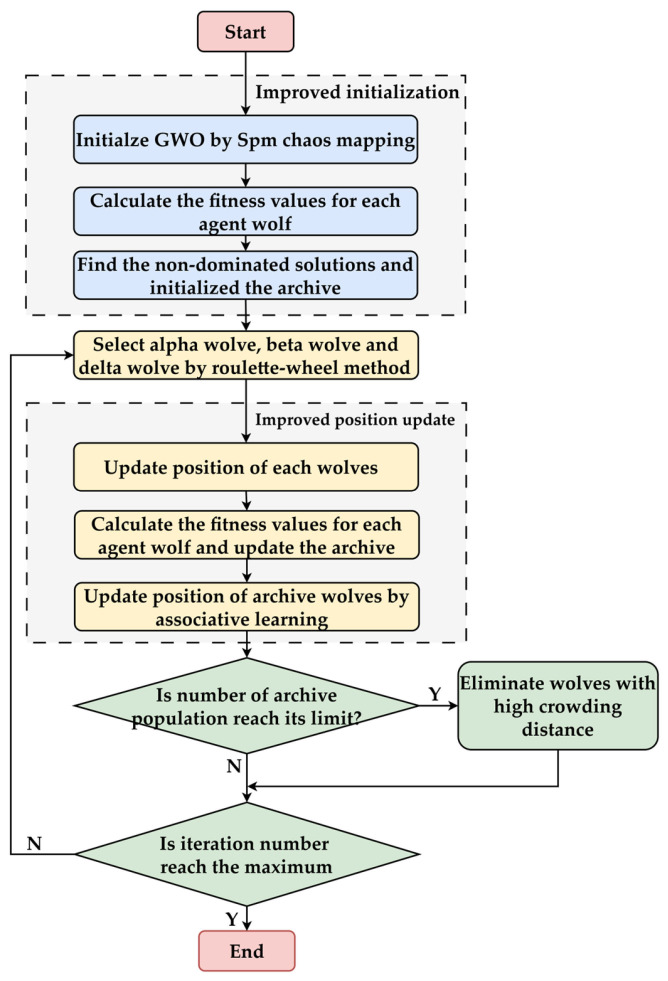
IMOGWO flowchart.

Figure 10 illustrates the flowchart of the proposed IMOGWO, which incorporates the Spm chaotic mapping initialization, an improved convergence coefficient, and an associative learning mechanism.

### 4.4. Modeling and Analysis of the IMOGWO

The aim of this paper is to minimize the maximum thinning rate, $\eta_{\max}$, and maximum geometric deviation, $L_{\max}$, during forming. Constraints include the tool radius, r, tool path strategy, b, initial sheet thickness, $t_{0}$, forming angle, θ, and step depth, Λ, all of which must remain within specified ranges. According to Table 1 and the established MLP model for predicting the SPIF thinning rate and geometric deviation, the mathematical optimization model for the IMOGWO can be formulated as follows:

(13) $$\begin{array}{cc} \underset{r,b,t_{0},\Lambda ,\theta }{\min} & \left(\begin{array}{c} \eta_{\max}\left(r,b,t_{0},\Lambda ,\theta \right)\\ L_{\max}\left(r,b,t_{0},\Lambda ,\theta \right) \end{array}\right)\\ s.t. & 2.5\leq r\leq 5\;\;r\in R\\ & b\in \left\{1,2,3\right\}\\ & 0.6\leq t_{0}\leq 1.2\;\;t_{0}\in R\\ & 0.125\leq \Lambda \leq 1\;\;\Lambda \in R\\ & 30^{{}^{\circ}}\leq \theta \leq 45^{{}^{\circ}}\;\;\theta \in R \end{array}$$

where R is the set of real numbers.

The wolf population size, maximum iterations, and Pareto archive capacity for IMOGWO were all set to 100. A key merit of the proposed IMOGWO lies in its minimal dependence on hyperparameter tuning. By restricting primary parameters to population size and iterations, the algorithm mitigates the performance fluctuations frequently observed in more parameter-intensive metaheuristics.

**Figure 11:**
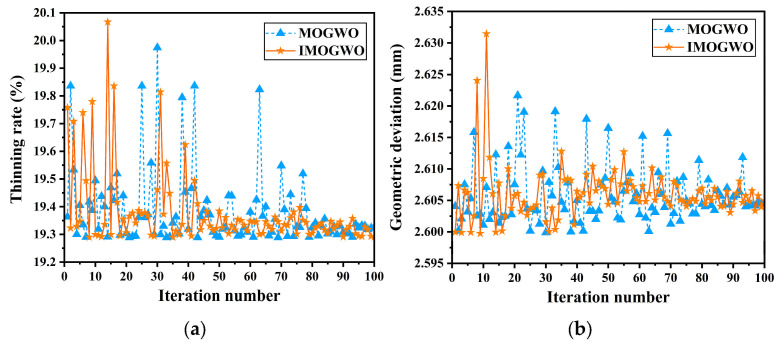
Comparison of the convergence processes of IMOGWO and MOGWO: (**a**) thinning rate; (**b**) geometric deviation.

The comparison of the convergence processes of IMOGWO and MOGWO is illustrated in Figure 11. As can be seen, IMOGWO converged at the 45th iteration, whereas MOGWO required 80 iterations, which demonstrates the superior convergence speed of IMOGWO. Upon convergence, IMOGWO maintained a maximum thinning rate of approximately 19.3% and a maximum geometric deviation of around 2.065 mm. This demonstrates that the proposed IMOGWO significantly reduces sheet thinning while preserving high forming performance.

**Figure 12:**
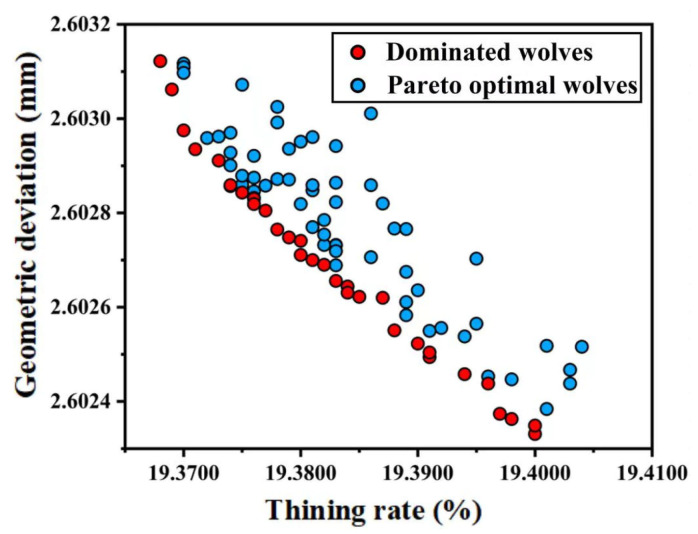
Pareto front.

Figure 12 shows the obtained Pareto front, which includes the identified 47 optimal parameter solutions (indicated by red dots). The target values of the Pareto optimal solutions are tabulated in Table 5.

To validate its effectiveness, the proposed IMOGWO was evaluated against MOGWO, NSGA-II, and the dynamical ensemble machine-learning-based MOGWO (DEML-MOGWO) [25]. To ensure a fair comparison, all algorithms employed the same population size (100) and maximum number of iterations (100). NSGA-II used a crossover probability of 0.9 and a mutation probability of 0.1. MOGWO and IMOGWO followed the standard parameter settings suggested in the original literature.

The performance of the various multi-objective optimization algorithms was evaluated quantitatively using hypervolume (HV) and spacing metric. HV measures the volume of the objective space dominated by the obtained Pareto front with respect to a reference point, defined as 1.1 times the maximum objective values found in the front, reflecting both convergence and solution diversity. Spacing evaluates the uniformity of the distribution of solutions along the Pareto front.

Table 6 summarizes the mean and standard deviation of the HV and spacing metric, calculated based on the final Pareto fronts from 30 independent runs. The proposed IMOGWO exhibited superior performance. It achieved the highest mean HV (7.50 × 10^−3^), surpassing NSGA-II (7.20 × 10^−3^), DEML-MOGWO (7.23 × 10^−3^) and the original MOGWO (7.02 × 10^−3^). Furthermore, the mean spacing of IMOGWO (0.19 × 10^−3^) is notably lower than that of MOGWO (0.50 × 10^−3^) and NSGA-II (0.55 × 10^−3^). This substantial reduction in spacing indicates that the non-dominated solutions obtained by IMOGWO are much more uniformly distributed along the Pareto front. Meanwhile, the spacing for IMOGWO (0.19 × 10^−3^) and DEML-MOGWO (0.18 × 10^−3^) was found to be highly comparable. This suggests that both frameworks have an equivalent capability to maintain a highly uniform distribution along the Pareto front. However, the fact that IMOGWO achieves this level of uniformity through its refined search mechanism—without the additional computational complexity of a dynamic ensemble surrogate—further highlights the efficiency and structural robustness of the proposed IMOGWO. These results indicate that IMOGWO provides superior convergence quality and a more uniform Pareto front distribution.

### 4.5. Optimal Solution Selection Based on Entropy-Weighted TOPSIS

Given the conflicting and constrained nature of the two optimization objectives of maximum thinning rate and maximum geometric deviation, the solutions on the Pareto optimal front are non-dominated trade-offs that cannot be compared directly. Consequently, the entropy-weighted TOPSIS method was employed to evaluate and rank the identified 47 Pareto optimal solutions. TOPSIS is one of the most widely employed multi-criteria decision-making approaches [38], ranking solutions based on their proximity to positive and negative ideal solutions. The optimal solution is the one that is closest to the positive ideal solution and furthest from the negative ideal solution. The entropy-weighted TOPSIS decision method improves decision accuracy by calculating the information entropy of different attributes to determine the weighting of process parameter evaluation indicators.

The IMOGWO has derived a Pareto front comprising two cost-type indicators: maximum SPIF thinning rate and maximum geometric deviation, both of which have lower values that are preferable. Furthermore, given the varying quantities and units of these attributes, normalization was required to prevent computational errors and distortion of the results. Consequently, a standardized decision matrix, ***Q***, can be constructed and expressed as Equation (14).

(14) $$Q={\left(q_{ij}\right)}_{n\times m}=\left[\begin{array}{ccc} \frac{1/y_{11}}{\sqrt{\sum_{i=1}^{n}{\left(1/y_{i1}\right)}^{2}}} & \ldots & \frac{1/y_{1m}}{\sqrt{\sum_{i=1}^{n}{\left(1/y_{i1}\right)}^{2}}}\\ \vdots & \ddots & \vdots \\ \frac{1/y_{n1}}{\sqrt{\sum_{i=1}^{n}{\left(1/y_{im}\right)}^{2}}} & \ldots & \frac{1/y_{nm}}{\sqrt{\sum_{i=1}^{n}{\left(1/y_{im}\right)}^{2}}} \end{array}\right]$$

where n and m represent the number of the Pareto optimal solutions and cost-type indicators (optimization objectives) within the Pareto front, respectively. $y_{ij}$ indicates the *j*-th indicator of the *i*-th solution on the Pareto optimal front. i=1,2,…,n and j=1,2,…,m.

The entropy value of the *j*-th indicator, $h_{j}$, can be calculated as follows:

(15) $$h_{j}=-\frac{1}{\ln n}\sum_{i=1}^{n}\left(\tau_{ij}\ln\tau_{ij}\right)$$

where $\tau_{ij}$ represents the normalized weight of the *j*-th indicator under the *i*-th solution, which is calculated as the ratio of its value to the sum of all indicator values for that solution:

(16) $$\tau_{ij}=\frac{q_{ij}}{\sum_{k=1}^{m}q_{ik}}$$

The entropy weight for the *j*-th indicator can be calculated as follows:

(17) $$\omega_{j}=\frac{1-h_{j}}{\sum_{k=1}^{m}\left(1-h_{k}\right)}$$

Combined Equations (14) and (17), the weighted normalized decision matrix, U, can be given as:

(18) $$U={\left(u_{ij}\right)}_{n\times m}=\left[\begin{array}{ccc} u_{11} & \cdots & u_{1m}\\ \vdots & \ddots & \vdots \\ u_{n1} & \cdots & u_{nm} \end{array}\right]=\left[\begin{array}{ccc} \omega_{1}q_{11} & \cdots & \omega_{m}q_{1m}\\ \vdots & \ddots & \vdots \\ \omega_{1}q_{n1} & \cdots & \omega_{m}q_{nm} \end{array}\right]$$

Considering that both the geometric deviation and the thinning rate should be minimized in SPIF processing, the positive and negative ideal solutions $S^{+}$ and $S^{-}$ can be expressed as:

(19) $$\left\{\begin{array}{l} S^{+}=[{s_{1}}^{+},{s_{2}}^{+},\ldots ,{s_{m}}^{+}]\\ S^{-}=[{s_{1}}^{-},{s_{2}}^{-},\ldots ,{s_{m}}^{-}] \end{array}\right.$$

where ${s_{j}}^{+}=\underset{i\in \left\{1,\ldots ,n\right\}}{\min}(u_{ij})$, ${s_{j}}^{-}=\underset{i\in \left\{1,\ldots ,n\right\}}{\max}(u_{ij})$.

Combining Equations (18) and (19), the Euclidean distances $d_{i}^{+}$ and $d_{i}^{-}$ of the *i*-th solution to the positive and negative ideal solutions, respectively, along with the resulting relative closeness, $C_{i}$, of the *i*-th solution to the positive ideal solution can be calculated as follows:

(20) $$\left\{\begin{array}{l} d_{i}^{+}=\sqrt{\sum_{j=1}^{m}{\left({s_{j}}^{+}-u_{ij}\right)}^{2}}\\ d_{i}^{-}=\sqrt{\sum_{j=1}^{m}{\left({s_{j}}^{-}-u_{ij}\right)}^{2}}\\ C_{i}=\frac{d_{i}^{-}}{d_{i}^{+}+d_{i}^{-}} \end{array}\right.$$

The relative closeness values of the Pareto optimal solutions are listed in Table 7. It can be seen that the optimal process parameters for SPIF were found to be those of solution 42, which exhibited the highest closeness (corresponding to a value of 0.995, highlighted in bold in the table). These parameters are: a tool radius of 4.00871 mm, an initial sheet thickness of 1.07692 mm, an axial step depth of 0.18797 mm, and a forming angle of 30°, with a contour path.

Physically, the optimized parameters reflect coupled trade-offs in SPIF mechanics. Step depth and tool radius jointly balance strain localization, thinning suppression, and geometric deviation, leading to interior optimal values. A larger initial sheet thickness increases load-bearing capacity and delays necking, but also raises forming forces and contact stresses. In contrast, forming angle exhibits a largely monotonic influence: a smaller angle reduces material stretching, membrane strain, and through-thickness thinning while improving geometric deviation. Consequently, the optimization naturally drives the forming angle to its lower bound, whereas the other parameters converge to interior values within the feasible design space.

## 5. Experimental Validation and Results Analysis

To evaluate the effectiveness of the proposed optimization framework, numerical simulations of SPIF for Al1060 truncated conical components were performed using parameters optimized by the IMOGWO, MOGWO, and NSGA-II algorithms in ABAQUS 2021 software. Subsequently, actual SPIF experiments were conducted using the optimal parameters obtained from the IMOGWO. The results of the experiments and simulations were then compared to verify the superiority of the proposed IMOGWO method.

### 5.1. Numerical Simulation and Results Analysis

To validate the performance of the proposed algorithm, the optimal combinations of process parameters for a truncated cone were solved using the proposed IMOGWO, traditional MOGWO, and NSGA-II algorithms, respectively. The ideal profile of the truncated cone has a top radius of 50 mm, a bottom radius of 7 mm, and a height of 25 mm. The predicted thinning rates and geometric deviation were calculated using the established MLP model integrated into three algorithms. The simulated thinning rates and geometric deviation were then calculated using the extracted numerical simulation results. Table 8 tabulates the optimal process parameters, as well as the predicted and simulated thinning rates and geometric deviation for the three algorithms. As can be seen in Table 8, the predicted and simulated thinning rates from the three algorithms are basically consistent. Among all algorithms, IMOGWO achieved the minimum thinning rates in the prediction (19.31%) and the simulation (18.550%). The predicted and simulated thinning rates of IMOGWO are 3.16% and 4.67% higher than those of MOGWO, and 6.71% and 7.73% higher than those of NSGA-II, respectively. Regarding geometric deviation, the predicted and simulated values from IMOGWO and MOGWO significantly outperformed those from NSGA-II. The predicted and simulated geometric deviation of IMOGWO are 20.56% and 21.78% higher than those of NSGA-II, respectively. These results demonstrate that the proposed IMOGWO is effective in the co-optimization of thinning rate and geometric deviation, suppressing excessive sheet thinning while ensuring forming quality.

**Figure 13:**
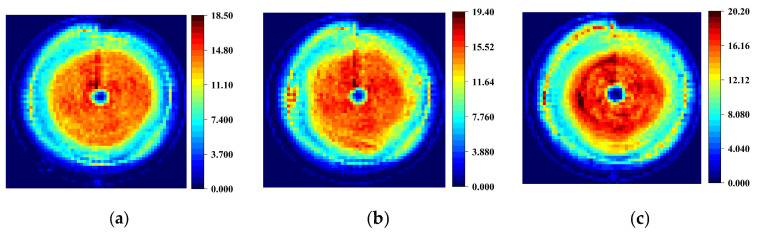
The heatmaps of thinning rates simulated under process parameters derived from three algorithms: (**a**) IMOGWO; (**b**) MOGWO; (**c**) NSGA-II.

To intuitively understand the effect of process parameters on sheet thinning, the heatmaps of thinning rates simulated under the parameters derived from three algorithms are presented in Figure 13. The color bars indicate the sheet thinning rate, with the rate increasing from blue to red. It can be concluded that a similar pattern emerges in all three maps: Overall, all three images exhibit the fundamental pattern of the lowest thinning rate at the central region, gradually increasing along the radial direction, and forming the maximum thinning region at the outer periphery. This is because during the initial forming stage, the material experiences strong constraints from the clamping end. The deformation mode is predominantly characterized by intense planar stretching, leading to significant stress concentration and strain localization, thereby forming the maximum thinning region. As the deformation mode gradually transitions from tensile-dominated to tensile-shear coupled, accompanied by compensatory material flow from the central region below, this inhibits further wall thinning when observed radially inward. Consequently, the thinning rate begins to decline. Subsequently, as the in-plane tensile strain increases to meet the geometric requirements of the component, the thinning rate increases steadily. Finally, at the center-bottom of the truncated cone at the forming endpoint, the deformation mode approximates pure bending with minimal in-plane tensile strain. Consequently, sheet thickness in this region remains essentially unchanged, reflecting the axisymmetric strain distribution characteristic of the SPIF forming process.

Figure 13 also demonstrates that the overall thinning in the IMOGWO simulation results is significantly reduced and more uniform. The annular thinning regions exhibit smooth, continuous transitions without local peaks or abnormal fluctuations. In contrast, the results from the MOGWO and NSGA-II algorithms show numerous high thinning rate points in the peripheral region and localized positions, indicating poorer uniformity in the distribution of thinning and suggesting certain local instabilities in the formation process.

**Figure 14:**
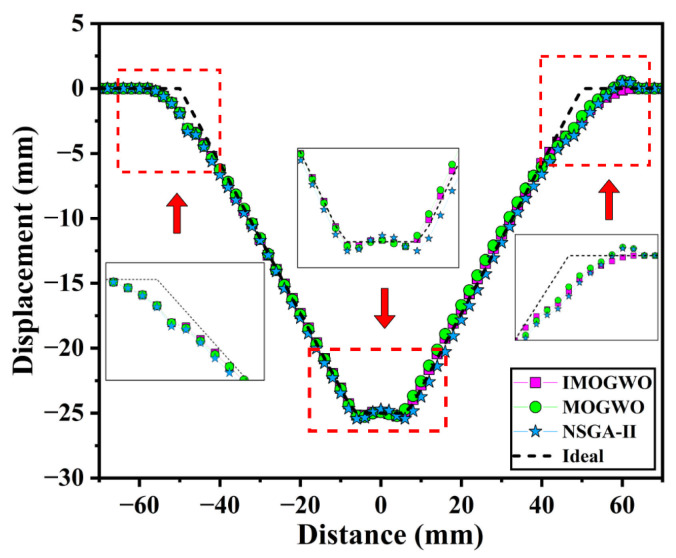
Comparison between the simulated and ideal profiles of truncated conical components under the process parameters obtained by the three algorithms. The red dashed boxes and arrows indicate magnified views of the entry regions and the bottom vertex to highlight geometric deviations and springback effects.

Figure 14 illustrates the simulated and ideal profiles obtained under the process parameters derived from the three algorithms. Overall, all three algorithms demonstrate satisfactory reconstruction of the ideal profile, with outward bulging observed during the initial forming stage. This phenomenon primarily occurs because the sheet metal has just entered the contact and plastic deformation phase at the onset of forming. The contact state remains unstable, local stiffness undergoes abrupt changes, and spring back exerts a significant influence, consequently reducing profile tracking accuracy. Furthermore, towards the end of the forming process, the combined effects of tool unloading, spring back and redistribution of residual stresses induce an upward arching deformation in the bottom region, resulting in the characteristic pillow-shaped profile.

As illustrated in Figure 14, the profile generated by the proposed IMOGWO is the most congruent with the ideal profile. The resulting curve is smoother, with significantly lower geometric deviations than those of MOGWO and NSGA-II. Notably, the transition region under IMOGWO demonstrates a more uniform variation, indicating a superior capability in suppressing the “convex” and “pillowing” effects during SPIF. Specifically, in the transition region, the maximum and mean deviations are 2.154 mm and 0.442 mm for IMOGWO, compared to 2.159 mm and 0.557 mm for MOGWO, and 2.754 mm and 0.712 mm for NSGA-II. These results validate that the proposed IMOGWO achieves optimal geometric deviation while being more stable against algorithmic oscillations and local parameter instability.

### 5.2. Experimental Results and Analysis

**Figure 15:**
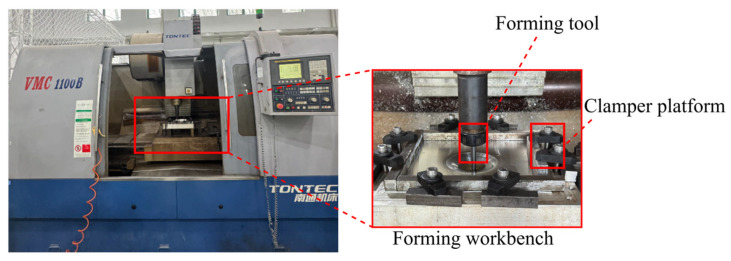
SPIF experimental apparatus.

To validate the reliability of the numerical simulation results and further assess the effectiveness of the proposed algorithm, an SPIF experiment on a truncated conical component was conducted in practice. A VMC1100B CNC machine manufactured by Nantong Machine Tool Co., Ltd. in Nantong, China, was employed as the forming platform, as illustrated in Figure 15. The forming tool was fabricated from high-hardness, heat-resistant tungsten high-speed steel. During the forming process, lithium-based grease was uniformly applied to the contact surface between the tool head and the aluminum sheet to reduce the coefficient of friction and enhance process stability. Considering the limitations of the experimental equipment in terms of hardware capabilities and machining precision, the actual forming process parameters were determined based on the optimal parameters obtained from IMOGWO: a tool radius of 4 mm, an initial sheet thickness of 1.1 mm, a step depth of 0.18797 mm, and a forming angle of 30°. During the forming process, the forming tool applied load layer by layer along a pre-set contour path to achieve continuous plastic deformation of the aluminum sheet. To minimize experimental error and suppress random noise, five replicate experiments were conducted under the optimal parameter combination, and the experimental results in this section are reported as the mean values of these five experiments. The mean thinning rate was 19.213 ± 0.072%, and the mean geometric deviation was 2.448 ± 0.015 mm, demonstrating low variability and good experimental repeatability.

**Figure 16:**
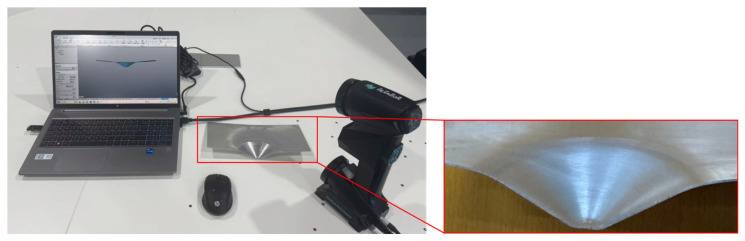
The truncated cone specimen obtained by wire cutting after SPIF forming, along with its profile measurements using a coordinate measuring machine.

The truncated cone specimen obtained via wire cutting after SPIF forming, along with its profile measurements using a coordinate measuring machine, is shown in Figure 16. The experimental truncated cone has an excellent overall forming quality. There are no surface fractures or significant geometric distortions. The outer surface is smooth with no pronounced tool marks, while the inner surface shows no tearing. This demonstrates that the chosen process parameters possess favorable forming adaptability and are capable of producing high-quality components.

**Figure 17:**
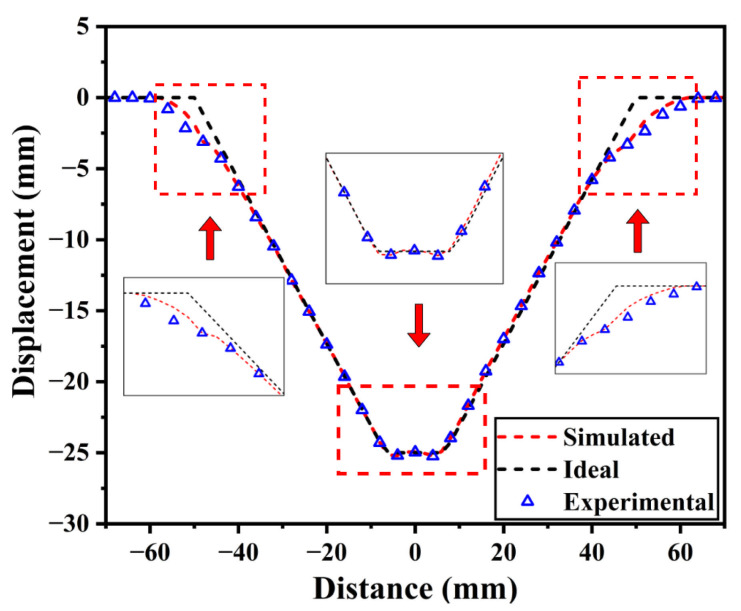
Comparison of experimental, simulated and ideal profiles. The red dashed boxes and arrows indicate magnified views of the entry regions and the bottom vertex to highlight geometric deviations and springback effects.

To accurately evaluate the geometric deviation of the formed cone, the experimentally scanned profile was compared with the simulated and ideal profiles. The results are shown in Figure 17. It can be observed that the experimental, simulated and ideal profiles exhibit high consistency in overall morphology, accurately characterizing the cone’s geometric features. This demonstrates that the established finite element model and the proposed method are valid and reliable. However, as observed in the partially enlarged views of Figure 17, both the simulated and experimental profiles show some degree of bulging and pillow effect compared to the ideal profile in the initial deformation region on both sides and in the bottom corner region. This is primarily due to the effect of material elastic spring-back after unloading and residual stress release on geometric deviation during the forming process.

The experimental and simulated results for the maximum geometric deviation are shown in Table 9. The maximum geometric deviations from the ideal profile are 2.372 mm and 2.448 mm for the simulated and experimental profiles, respectively. The relative error of the simulated deviation with respect to the experimental value is 3.10%. This demonstrates that the IMOGWO method proposed in this paper can effectively reduce the geometric deviation of the formed parts for SPIF.

**Figure 18:**
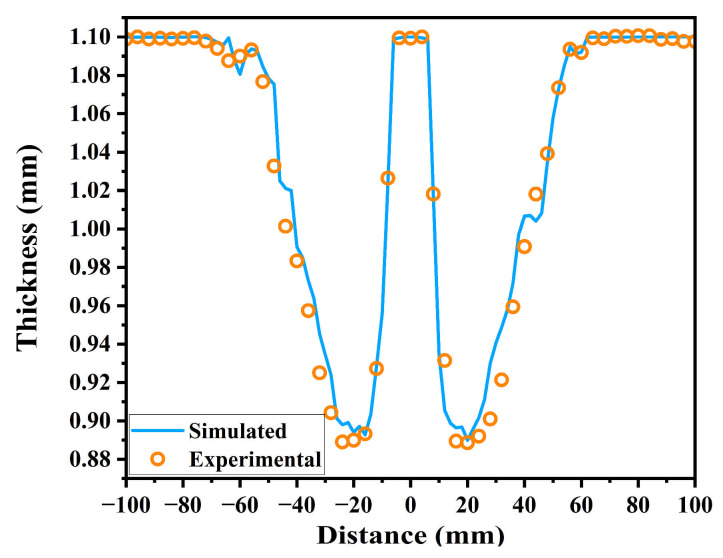
The experimental and simulated thicknesses for the truncated cone formed by SPIF.

To understand the thickness variation in the sheet after forming, 50 equidistant points were selected at 4 mm intervals along the wire cutting direction. The corresponding thickness at each point was then measured using a micrometer. The experimental and simulated thicknesses for the truncated cone formed by SPIF are shown in Figure 18. The experimental and simulated thicknesses are consistent, with the thickness remaining largely unchanged in the non-forming region and exhibiting significant thinning in the forming region. The minimum thicknesses for the experiment and the simulation are 0.8887 mm and 0.8899 mm, respectively, occurring in the final forming region near the location of the pillow effect. The corresponding thinning rates are 19.213% and 19.101%. This phenomenon arises because the undeformed bottom region experiences intense traction from the surrounding deformed region. Consequently, the transition region between the side walls and the bottom surface undergoes severe radial tensile stress, causing the material to thin dramatically before reaching the bottom plane.

The experimental and simulated results for the maximum thinning rate are also presented in Table 9. While maintaining low geometric deviation, it can be observed that the experimental and simulated thinning rates are 5.815% and 5.703% higher than the rate calculated by the sine law, respectively. However, the overall discrepancy remains small, falling within an acceptable range, with a relative error of merely 0.58% between the simulated and experimental thinning rates. This indicates that the proposed IMOGWO algorithm is effective in minimizing excessive thinning. It should be noted that the sine law is based on ideal film theory and does not account for the physical effects that are common in SPIF processes, such as bending-tension coupled deformation, localized three-dimensional stress states, friction, and material hardening. To reduce the geometric deviation, the optimization algorithm strengthens local forming stability, thereby increasing plastic strain accumulation and thickness reduction to some extent. Consequently, the deviations between the experimental and simulated results and the sine law prediction are both expected and inevitable, given the latter’s idealized assumption. Therefore, it can be concluded that the proposed IMOGWO algorithm is effective and feasible for co-optimizing geometric deviation and thinning rate in SPIF.

## 6. Conclusions

This study developed a dual-objective optimization framework that integrates an IMOGWO with an MLP to synergistically optimize the thinning rate and geometric deviation in SPIF. The global search capability and convergence efficiency of the algorithm were improved by incorporating the Spm chaotic mapping, an improved convergence coefficient, and an associative learning mechanism. An entropy-weighted TOPSIS method was employed successfully to identify the optimal process parameters from the Pareto front. The simulation and experimental testing were conducted on a truncated cone component using the solved optimal process parameters. The simulation results indicate that the maximum thinning rate and geometric deviation of the proposed IMOGWO method are 4.67% and 0.23% higher than those of MOGWO, and 7.73% and 21.78% higher than those of NSGA-II, respectively. Furthermore, the experimental results show that the maximum thinning rate of the formed truncated cone is 19.213%, with a maximum geometric deviation of 2.448 mm. The simulated and experimental results are highly consistent, confirming the effectiveness and feasibility of the proposed IMOGWO method to solve the bi-objective optimization problem of thinning rate and geometric deviation in SPIF. This study provides a robust and practical solution for the multi-objective intelligent optimization in SPIF, effectively balancing thinning rate constraints with geometric deviation requirements.

## 7. Limitations and Future Work

The proposed IMOGWO-MLP framework may exhibit reduced effectiveness or failure under several conditions: insufficient surrogate model fidelity due to sparse or unrepresentative training data; severe extrapolation beyond the sampled design space; highly unstable or bifurcation-prone forming regimes such as wrinkling, tearing, or localized necking; strongly stochastic or poorly controlled industrial conditions; or inaccurate constitutive models that fail to capture material behavior. Moreover, applying the framework to non-axisymmetric, multi-feature components and alternative materials requires high-fidelity finite element simulations and accurate large-strain constitutive models that account for plasticity, hardening, and anisotropy. It also requires sufficient MLP surrogate capacity to capture higher-order nonlinearities and strong parameter coupling. In such cases, increased computational cost and reduced surrogate accuracy due to limited training data may further constrain its practical applicability.

Future work will focus on improving the robustness, generality, and industrial applicability of the proposed IMOGWO-MLP framework by incorporating uncertainty quantification, adaptive modeling strategies, and extended quality indicators.

1. While the current IMOGWO-MLP framework provides significant numerical improvements, the current analysis assumes deterministic process conditions. Future research will explore the sensitivity and robustness of these optimal solutions under stochastic process variations to ensure consistent performance in industrial environments.
2. Future research will focus on extending the framework to more complex geometric configurations and material systems to enable its reliable application to non-axisymmetric, multi-feature components and a broader range of materials.
3. Recent studies have shown that microstructural evolution and crystallographic texture play an important role in thinning, formability, and anisotropy in SPIF. These effects are not considered in the present work. Future studies will focus on the coordination between macroscopic process parameters and microscopic material behavior, aiming to develop a coupled macro-micro framework to improve the reliability and applicability of the proposed method across different materials and forming conditions.
4. While the study offers a robust contribution to understanding process optimization for thinning rate and geometric deviation in SPIF, certain aspects warrant further exploration. Future research will incorporate surface roughness, forming limit proximity through repeated trials and probabilistic simulations.

## Figures and Tables

**Table 1:** SPIF process parameters and their levels.

Parameter	Levels				
Tool radius (mm)	2.5	3	4	5	-
Initial sheet thickness (mm)	0.6	0.8	1	1.2	-
Tool path strategy	Contour	Helical	Reverse contour	-	-
Step depth (mm)	0.125	0.25	0.5	0.75	1
Forming angle (°)	30	40	45	-	-

**Table 2:** Chemical composition of Al1060.

Element	Fe	Si	Cu	Zn	Mg	Mn	V	Ti	Al
Content (%)	0.35	0.25	0.05	0.05	0.03	0.03	0.05	0.03	99.6

**Table 3:** The primary physical and mechanical properties of Al1060.

Properties	Symbol	Value
Density (kg/m^3^)	ρ	2700
Young’s modulus (GPa)	E	70
Poisson’s ratio	μ	0.33
Yield stress (MPa)	$\sigma_{y}$	136
Ultimate tensile stress (MPa)	$\sigma_{u}$	162
Elongation (%)	l	24.9
Saturation flow stress (MPa)	$\sigma_{0}$	163.94
Coefficient of Voce equation	A	0.162
	B	9.42

**Table 4:** The performance comparison of different MLP architectures based on five-fold cross-validation.

Model	Hidden Layers	Neurons Setting	Mean Thinning Rate		Mean Geometric Deviation	
			*MSE* (%^2^)	*R* ^2^	*MSE* (mm^2^)	*R* ^2^
Adopted MLP	5	(100, 80, 60, 40, 20)	0.0406	0.924	0.0438	0.935
MLP-1	5	(20, 20, 20, 20, 20)	0.0558	0.858	0.0596	0.890
MLP-2	3	(100, 60, 20)	0.0556	0.896	0.0504	0.914
MLP-3	3	(20, 20, 20)	0.0732	0.877	0.0679	0.885

**Table 5:** Target values of the Pareto optimal solutions.

Proposal	$\eta_{\max}$ (%)	$L_{\max}$ (mm)
1	19.3636	2.6031
2	19.3646	2.6029
3	19.3648	2.6029
……	……	……
47	19.3684	2.6023

**Table 6:** Quantitative performance comparison of multi-objective optimization algorithms.

Algorithm	Hypervolume (Mean ± Std)	Spacing (Mean ± Std)
IMOGWO	(7.50 ± 0.01) × 10^−3^	(0.19 ± 0.01) × 10^−3^
MOGWO	(7.02 ± 0.02) × 10^−3^	(0.50 ± 0.02) × 10^−3^
NSGA-II	(7.20 ± 0.01) × 10^−3^	(0.55 ± 0.01) × 10^−3^
DEML-MOGWO	(7.23 ± 0.01) × 10^−3^	(0.18 ± 0.02) × 10^−3^

**Table 7:** The relative closeness values of the Pareto optimal solutions.

Proposal	Tool Radius (mm)	Initial Sheet Thickness (mm)	Tool Path Strategy	Step Depth (mm)	Forming Angle (°)	Closeness
1	3.90368	1.09945	Contour	0.19257	30	0.60919
2	3.89689	1.10173	Contour	0.18966	30	0.60939
			…			
42	4.00871	1.07692	Contour	0.18797	30	0.99500
			…			
47	3.84018	1.09292	Contour	0.19506	30	0.20353

**Table 8:** Optimal process parameters, as well as the predicted and simulated thinning rates and geometric deviation for the three algorithms.

Optimization Algorithm	Tool Radius (mm)	Initial Sheet Thickness (mm)	Tool Path Strategy	Step Depth (mm)	Forming Angle (°)	$\eta_{\max}$ (%)	$L_{\max}$ (mm)	$\eta_{\max}$ (%)	$L_{\max}$ (mm)
						Predicted		Simulated	
IMOGWO	4.0087	1.0769	Contour	0.18797	30	19.31	2.0654	18.550	2.154
MOGWO	3.6841	1.0518	Contour	0.1946	30	19.94	2.0643	19.459	2.159
NSGA-II	2.6506	1.1825	Contour	0.1250	30	20.70	2.5998	20.104	2.754

**Table 9:** The experimental and simulated results for thinning rate and geometric deviation.

	Experimental	Simulated	Sine Law
$\eta_{\max}$ (%)	19.213	19.101	13.398
$L_{\max}$ (mm)	2.448	2.372	-

## Data Availability

The original contributions presented in this study are included in the article. Further inquiries can be directed to the corresponding author.
